# Sagittal component alignment is less reliable than coronal component alignment in a Chinese population undergoing navigated TKA

**DOI:** 10.1186/s13018-014-0051-1

**Published:** 2014-07-06

**Authors:** Xiaoyong Chen, Huayi Wang, Yuanzhen Cai, Qingsheng Zhu, Jinyu Zhu

**Affiliations:** 1 Department of Orthopedics, Xijing Hospital, Fourth Military Medical University, Xi’an 710032, China; 2 Department of Orthopedics, Chenggong Hospital affiliated to Xiamen University, Xiamen 361000, China

**Keywords:** Total knee arthroplasty, Computer-assisted, Sagittal alignment, Navigation

## Abstract

**Background:**

The purpose of our study was to determine whether postoperative sagittal component alignments of primary total knee arthroplasty (TKA) using the conventional and navigated technique differed significantly. Additionally, we determined whether the use of navigation systems resulted in hyperextension of the femoral components in Chinese patients.

**Methods:**

This retrospective study reviewed 36 consecutive patients (72 knees) who underwent simultaneous bilateral primary TKAs at our hospital from February 2011 to March 2012. One knee was replaced using a computer-assisted navigation system, and the contralateral knee was replaced with the conventional technique. The radiographic and clinical results of both groups were compared. The relationship between preoperative anatomic angles and component alignments in conventional TKA and navigated TKA was examined.

**Results:**

The radiographic results showed statistically significant differences only between the navigated and conventional groups for individual femoral coronal and sagittal component alignment. Femoral sagittal component alignment showed less deviation and tended to have hyperextension using the navigated technique (−0.35°) compared with the conventional technique (2.77°). There was no significant difference observed for the Knee Society Score (KSS) between the two groups at 2 years postoperatively.

**Conclusions:**

The sagittal component alignment of primary TKA obtained using the conventional and navigated techniques differed significantly. Navigated TKAs resulted in a higher risk of hyperextension of the femoral components in Chinese patients.

## Background

Computer navigation systems are designed to increase the accuracy and consistency of prosthetic alignment in total knee arthroplasty (TKA). Many studies have shown a reduction in the number of outliers, that is, misalignments of >3°, in both the components and the lower limb mechanical axis using the navigated technique [[1]–[6]]. The accuracy of prosthetic alignment in the sagittal plane has been less emphasised in previous studies compared to coronal component alignment.

Sagittal alignment of the femoral component may influence the clinical results of TKA in various ways. If a femoral component is placed in hyperflexion, the extension or polyethylene post wear resulting from impingement between the anterior part of the polyethylene insert and the intercondylar box of the femoral component in TKA can be limited [[7]]. When a femoral component is placed in hyperextension relative to the femur, it may create a notch in the anterior femoral cortex, which can increase the potential risk of a supracondylar fracture [[8],[9]]. Based on these data, ignorance of sagittal component alignment in TKA is not acceptable. A previous study examining the use of standing radiographs of the entire lower extremity has shown that targeted sagittal component alignments of TKA achieved using the conventional and navigated techniques differed significantly. The use of navigation systems resulted in hyperextension between the femoral and tibial components [[10]]. However, the subjects included in the previous study were healthy volunteers without symptoms or radiographic abnormalities. Thus, these results may not be directly applicable to patients with lower limb deformity. In the Chinese population, bowing of the femur is commonly found in patients with osteoarthritis of the knee. The incidence of femur bowing is approximately 62% [[11],[12]].

The purpose of our study was to determine whether postoperative sagittal component alignments of simultaneous bilateral primary TKAs obtained using the conventional and navigated techniques differed significantly and whether the use of navigation systems resulted in hyperextension of the femoral components in Chinese patients undergoing navigated TKAs.

## Patients and methods

### Demographics

We retrospectively reviewed the hospital records of 40 consecutive patients (80 knees) who underwent simultaneous bilateral primary TKAs at our hospital from February 2011 to March 2012. One knee was replaced using a computer-assisted navigation system. The contralateral knee was replaced with a conventional technique using an intramedullar rod for the femur and a mechanical extramedullar guiding system for the tibia. The indications for surgery were rheumatoid arthritis and knee osteoarthritis. There were two patients with bilateral valgus knees and one patient with a unilateral valgus knee, who were excluded from the analysis. Furthermore, one (2.5%) patient was lost during the follow-up. In total, 36 patients with 72 TKAs were successfully followed up for more than 24 months (Table 1).

**Table 1:** Patient demographics

	**Total**
Number of patients	40
Gender	
Male	6
Female	30
Aetiology	
Rheumatoid arthritis	2
Osteoarthritis	34
BMI^a^ (kg/m^2^)	25.2 ± 4.6
Age^a^ (years)	61.3 ± 9.5

^a^The values are given as the mean and the standard deviation.

There were 30 female and 6 male patients. The sex distribution of arthritis is a common finding in a Chinese ethnic group. There were no significant differences in the preoperative anatomic angles and Knee Society Score (KSS) (Table 2).

**Table 2:** Preoperative radiographical and clinical measurements

	**Navigation**	**Convention**	***p*** **value**
Mechanical axis	9.69° ± 5.49°	9.45° ± 5.20°	0.620
Femoral anatomic valgus	6.56° ± 2.68°	6.66° ± 2.77°	0.627
Femoral anatomic flexion	4.03° ± 1.44°	4.15° ± 1.51°	0.336
Knee score	34.97 ± 10.16	34.36 ± 10.17	0.063
Function score	45.28 ± 11.08	44.03 ± 12.06	0.141

There was no significant difference between two groups (*p* > 0.05).

### Surgical techniques

All knees were implanted with NexGen posterior-stabilised total knee prosthesis (Zimmer, Warsaw, Indiana). The Stryker Precision Knee navigation-assisted system (Stryker-Leibinger, Freiburg, Germany) was employed. All surgeries were performed by two surgeons (QZ and JZ) experienced in using the Zimmer NexGen prosthesis. The procedure was performed through a midline skin incision of 10 to 12 cm in length with the use of a medial parapatellar arthrotomy. In the navigated cohort, the surgeon dialled for correct orientation of the jig according to the data presented by the navigation system. Once in position, the surgeon assured a stabile cut block fixation by tightening with pins. All resections were performed manually and verified by the universal tracker. In the conventional cohort, extramedullary instrumentation was used for the tibial component, and intramedullary instrumentation was used for the femoral side. After the bone resection in all knees, the contracted amount of soft tissue was carefully evaluated, and selective release was performed as required. The same protocol for postoperative management was utilised in both groups, which included bedside continuous passive motion machine therapy, physical therapy with partial weight bearing, and quadriceps and hamstring strengthening exercises starting on the second postoperative day.

### Radiological evaluation

**Figure 1:**
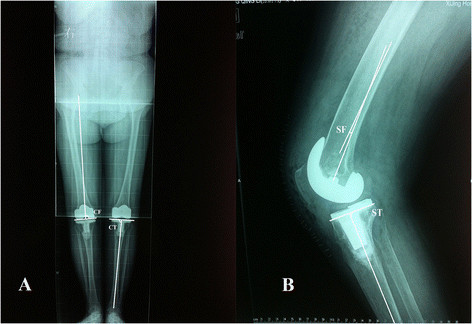
**Measurement of various angles on anteroposterior (A) and lateral (B) radiographs after total knee arthroplasty.** The component alignments were evaluated by measuring four modified angles including the coronal femoral angle (*CF*), coronal tibial angle (*CT*), sagittal femoral angle (*SF*) and sagittal tibial angle (*ST*).

Standard anteroposterior and lateral long-leg standing X-rays were obtained before and 6 weeks after surgery to determine the following parameters: the coronal lower-limb mechanical axis angle (MA) between the coronal femoral mechanical axis and the coronal tibial mechanical axis, the femoral anatomic valgus angle (FAV) between the coronal femoral mechanical axis and the distal femoral anatomic axis, and the femoral anatomic flexion angle (FAF) between the sagittal femoral mechanical axis and the femoral anatomic axis. As described by Hsu et al. [[13],[14]], the component alignments were evaluated by measuring four modified angles, i.e., (1) the coronal femoral angle (CF) between the mechanical axis of the femur and the transcondylar line of the femur as measured on the medial side, (2) the coronal tibial angle (CT) between the mechanical axis of the tibia and the tibial base plate as measured on the medial side, (3) the sagittal femoral angle (SF) between the sagittal femoral mechanical axis and the perpendicular axis of the femoral component and (4) the sagittal tibial angle (ST) between the sagittal tibial mechanical axis and the horizontal axis of the tibial component (Figure 1). The target values for implantation were recommended by the manufacturer: a MA of 0°, a CF of 90° for femoral component and a CT of 90° for the tibial component in the coronal plane. In the sagittal plane, the target value was an SF of 0° for the femoral component and a ST of 85° for the tibial component. The values of the mechanical axes with varus deformity were recorded as positive values. The goal of TKA implantation was to achieve a postoperative correction in the mechanical axis within a range of 3° varus or valgus. A radiological evaluation was performed by two independent observers (YC and HW). The results are expressed as the mean, range and standard deviation.

### Clinical evaluation

All patients were routinely assessed before surgery and then 6 months and annually after surgery. The KSS score was used for clinical evaluation [[15],[16]]. The KSS score is divided into the knee score and the function score. The patients underwent clinical evaluation at the outpatient clinic. Whenever it was not possible to come to the outpatient clinic (mainly because of transportation problems), these patients were visited by the examiner of a local hospital and were interviewed by telephone questionnaire. This was in agreement with the literature that supported the quality of data obtained using the telephone method [[16]].

### Statistical analysis

Statistical analyses were performed using SPSS 20 (SPSS Inc., Chicago, IL, USA) software. The preoperative data of both groups were compared using Student's *t* test. The radiographic and clinical results of both groups were compared using Student's *t* test and chi-square test. The relationship between preoperative anatomic angles and component alignments in conventional TKA and navigated TKA was examined using the Spearman correlation coefficient. The *p* value <0.05 indicates a statistically significant difference.

## Results

All patients were followed up for an average of 30.5 months (range, 24–36 months). There was no significant difference in postoperative complications between the two groups. There was one case of deep vein thrombosis in the navigated group and one case of serious bruises in the conventional group. One patient with bilateral knees required manipulation under anaesthesia because of knee stiffness. There were no patients who required additional surgery due to implant failure during the follow-up period. Surgery using the navigated technique lasted an average of 92 min for a TKA. Surgery using the conventional technique lasted an average of 60 min (*p* < 0.001).

The postoperative lower-limb alignment demonstrated more varus in the conventional group (2.25° ± 3.14°) than in the navigated group (1.19° ± 1.56°) (*p* = 0.028). There was a significant difference between these two measures with regard to the number of knees outside ±3° of a neutral mechanical axis (*p* = 0.032). The navigated group had 8.3% (3/36) of knees outside ±3° of neutral, whereas the conventional group had 27.8% (10/36). Additional alignment data can be found in Table 3. The femoral components in the conventional group (89.25° ± 3.32°) tended to be more varus than in the navigated group (90.60° ± 1.75°) (*p* = 0.014). In the sagittal plane, the femoral components inserted with navigation tended toward hyperextension (−0.35° ± 1.45°) vs. flexion (2.77° ± 2.21°) in the conventional group (*p* < 0.001). Similarly, the femoral components in the navigated group demonstrated less deviation from the femoral mechanical axis (*p* = 0.003). Simultaneously, the tibial component alignment tended to be in slight varus and flexion in both groups, and there was no significant difference.

**Table 3:** Postoperative radiographical measurements and outliers of lower-limb and component alignments

	**Measurements**			**Outliers**		
	**Navigation**	**Convention**	***p*** **value**	**Navigation (%)**	**Convention (%)**	***p*** **value**
Mechanical axis	1.19° ± 1.56°	2.25° ± 3.14°	0.028	8.3	27.8	0.032
Coronal femoral angle	90.60° ± 1.75°	89.25° ± 3.32°	0.014	25.0	50.0	0.028
Coronal tibial angle	88.21° ± 1.40°	88.50° ± 1.40°	0.294	33.3	25.0	0.437
Sagittal femoral angle	−0.35° ± 1.45°	2.77° ± 2.21°	<0.001	5.6	36.1	0.003
Sagittal tibial angle	85.77° ± 1.43°	85.14° ± 1.64°	0.069	1.4	16.7	0.743

An average value ± standard deviation is reported for each index.

There was a correlation (*r* = −0.754, *p* < 0.001) between the femoral anatomic valgus angle and postoperative coronal femoral angle in the conventional group. However, no correlation was found in the navigated patients. Similarly, there was a positive correlation (*r* = 0.836, *p* < 0.001) demonstrated between the femoral anatomic flexion and postoperative sagittal femoral angle in the conventional group. There was no correlation found in the navigated group (Figure 1).

The KSS scores are shown in Table 4. The knee score and function score were not different between the navigated and conventional groups (73.83 ± 9.84 vs. 75.14 ± 9.74 and 73.89 ± 10.69 vs. 72.78 ± 11.18) at 6 months postoperatively. There was no significant difference of KSS between the two groups at 2 years postoperatively.

**Table 4:** Postoperative clinical results at 6 months and 2 years

	**6 months**			**2 years**		
	**Navigation**	**Convention**	***p*** **value**	**Navigation**	**Convention**	***p*** **value**
Knee score	73.83 ± 9.84	75.14 ± 9.74	0.285	84.89 ± 7.85	85.28 ± 8.39	0.224
Function score	73.89 ± 10.69	72.78 ± 11.18	0.210	83.06 ± 9.43	82.08 ± 10.31	0.255

An average value ± standard deviation is reported for each index.

## Discussion

The most important finding of the present study was that postoperative sagittal component alignments of simultaneous bilateral primary TKA obtained using the conventional and navigated techniques differed significantly. Notably, the use of navigation systems resulted in hyperextension of the femoral components in Chinese patients undergoing TKAs.

Previous randomised control trials comparing conventional total knee arthroplasty with computer-assisted surgery have demonstrated a smaller range of deviation from all component alignments and fewer outliers with computer-assisted surgery [[17]–[22]]. However, other studies have failed to show a significant difference [[23],[24]]. In this comparative study, there were no significant differences in most variables for patients who underwent simultaneous bilateral primary TKAs. The evaluations of the radiographic results showed statistically significant differences only between the navigated and conventional groups for the femoral component. The overall percentage of TKAs that achieved the target component alignment in the femoral valgus and flexion angles was also higher with the navigated technique.

**Figure 2:**
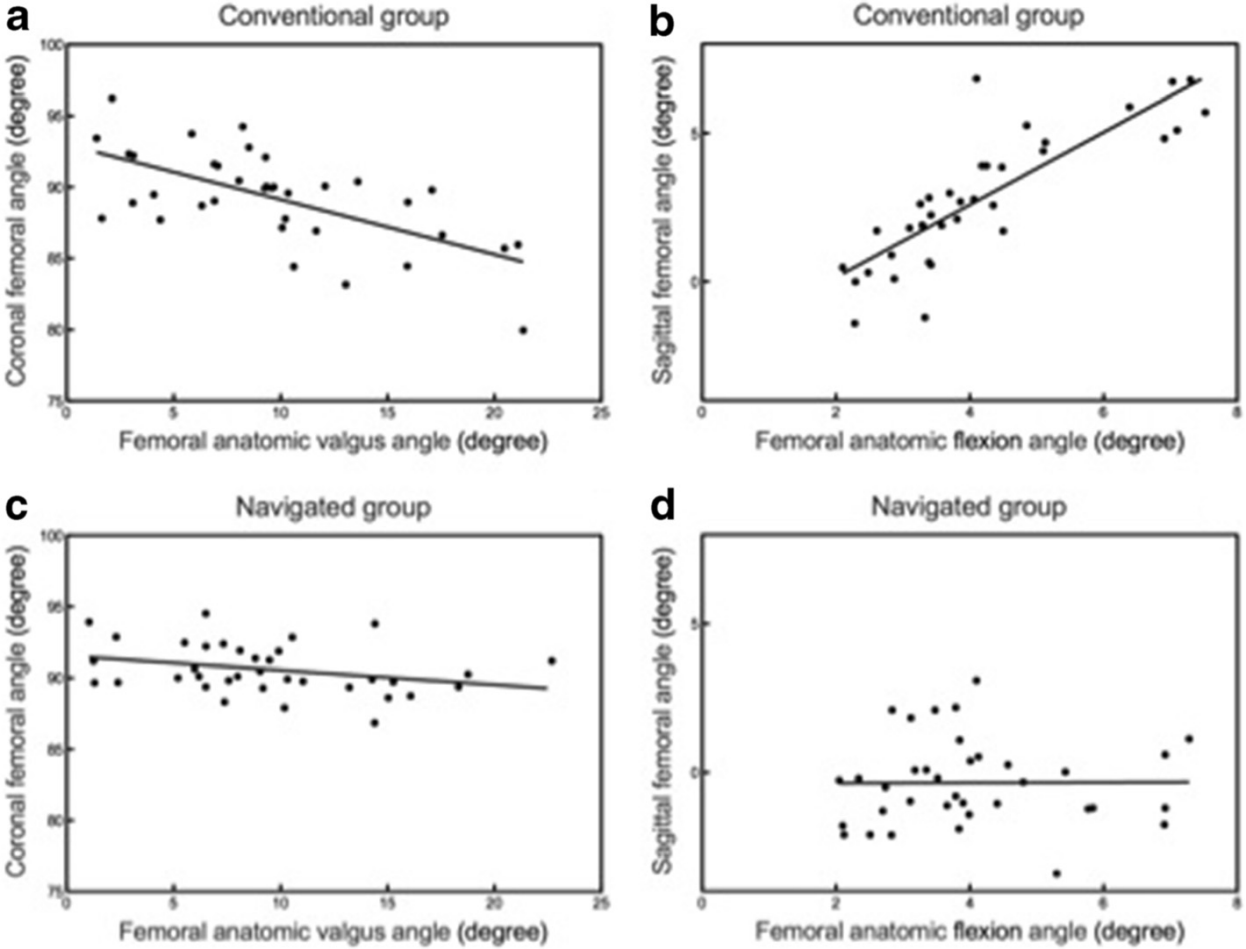
**Scatter plot showing a linear regression analysis of conventional group (a, b) and navigated group (c, d).** The preoperative femoral anatomic angle influenced the postoperative femoral component angle in conventional TKA, whereas no such correlation was found in the navigated patients.

In our study, postoperative femoral component alignment in the sagittal plane using the conventional and navigated techniques differed significantly. However, it was inconclusive whether sagittal component alignment after navigated surgery was significantly better than the conventional technique. The sagittal femoral component alignment showed less deviation and tended to be in hyperextension using the navigated technique compared with the conventional technique. The angles were −0.35° in the navigated group and 2.77° in the conventional group. Femoral component flexion allows for impingement of the femoral cam on the anterior aspect of the tibial post leading to anterior post wear and deformation, which can cause an increase in rotational constraint in knee extension. The hyperextension position of the femoral components increases the potential risk of osteolysis and anterior tibial post impingement with posterior stabilised prostheses [[25]]. Therefore, sagittal femoral component alignment in navigated TKA is less reliable than coronal component alignment. Although this parameter has not often been described in prior studies, the outcome was not unpredictable because the navigated technique does not account for the anterior bow of the femur. The centre of the femoral head and the distal femoral inputs tend to extend the femoral position. However, femoral bowing changes the angular relationship between the anatomical axis and the mechanical axis of the distal femur. This relationship determines the choice of the femoral cutting block and the distal femoral bone cut when using the conventional technique (Figure 2). If an intramedullary guide rod is inserted, the rod will follow the alignment of the distal femur. Because of the femoral bowing, conventional technique can never make the longitudinal axes of the implant and the femur parallel to each other. We recommend that it is important to modify the flexion angle of the femoral component suggested by the navigation software according to the femoral bowing. Anatomically, registration using an imageless bone morphing technique requires precise and widespread contact with the anterior femoral cortex by the navigation pointer to achieve sufficient information about distal femoral flexion and curvature. However, there is typically a significant amount of soft tissue on the anterior femur, and bone morphing may be impaired or inaccurate. We suggest that this is the source of the relative inaccuracy in the navigated knees.

Although this study reported no significant difference with tibial component alignments between navigated surgery and conventional surgery, there was a trend toward lower percentages of patients with misalignment outside of the acceptable range after navigated surgery. We suggest that the tibial coronal cut is a critical step. All subsequent femoral cuts and soft tissue balancing are based on the accuracy of this initial step. Furthermore, tibial loosening appears to be the most frequent fixation failure leading to early revision surgery [[26]–[29]]. Accordingly, the superior results associated with navigation relative to the tibial coronal cut are important to note.

Although the overall percentage of TKAs that achieved the target component alignments was higher with the navigated technique, there were no differences in postoperative KSS scores. There was no clear short-term clinical benefit to navigated TKA identified in this study. There may be additional technical factors besides lower-limb mechanical alignment contributing to the pattern of outcomes noted. These factors include soft tissue balance and joint line location [[30]], which are not only difficult to assess but challenging to accurately quantify. A dynamic gait pattern is also emerging as a possible important driver of outcome after TKA. Therefore, postoperative functions of TKAs are determined by multiple factors. There is still a potential risk that the importance of the mechanical alignment of the lower limb is overemphasised, and the role of other factors is ignored.

Several limitations in this study should be acknowledged. We did not randomise our patients. However, to limit selection bias, we reviewed consecutive patients who underwent simultaneous bilateral primary TKAs, which eliminated or reduced the effects of confounders. Additionally, it was relatively accurate to evaluate postoperative pain score by comparing bilateral knee joints for every patient. Another potential bias of the study is that the use of navigated or conventional TKA on the first knee may have provided information in planning for the contralateral knee. Second, a major limitation is that we did not use three-dimensional CT scans, which may have revealed differing results with regard to the radiographic outcomes assessed such as rotational positions of the femoral and tibial components. However, there were no intraoperative or postoperative patella complications and no insufficiencies of the knee extensor mechanism. In addition, it is difficult to perform CT while a subject is in the standing position. Third, the study lacked blinding of the radiographic review. It is difficult to perform a blinded review because the radiographic features of the pin tracts for the navigation arrays are often present.

## Conclusions

In summary, our retrospective, self-control study showed postoperative sagittal component alignments of simultaneous bilateral primary TKA obtained using the conventional and navigated techniques differed significantly. Using the navigated technique resulted in a higher risk of hyperextension of the femoral components compared to the conventional technique in Chinese patients undergoing TKAs.

## Competing interests

The authors declare that they have no competing interests.

## Authors' contributions

XC participated in the design of the study and drafted the manuscript. JZ and QZ participated in the design of the study and coordination and helped draft the manuscript. YC and HW participated in the radiological evaluation and performed the statistical analysis. All authors read and approved the final manuscript.
